# Gastrointestinal Tract Disorders in Older Age

**DOI:** 10.1155/2019/6757524

**Published:** 2019-01-17

**Authors:** Igor Dumic, Terri Nordin, Mladen Jecmenica, Milica Stojkovic Lalosevic, Tomica Milosavljevic, Tamara Milovanovic

**Affiliations:** ^1^Division of Hospital Medicine, Mayo Clinic Health System, Eau Claire, WI, USA; ^2^Mayo Clinic College of Medicine and Sciences, Rochester, MN, USA; ^3^Department of Family Medicine, Mayo Clinic Health System, Eau Claire WI, USA; ^4^Gastroenterology Fellowship Program, The Wright Center for Graduate Medical Education, Scranton, PA, USA; ^5^Clinic for Gastroenterology and Hepatology, Clinical Center of Serbia, Belgrade, Serbia; ^6^School of Medicine, Belgrade University, Belgrade, Serbia

## Abstract

Considering an increase in the life expectancy leading to a rise in the elderly population, it is important to recognize the changes that occur along the process of aging. Gastrointestinal (GI) changes in the elderly are common, and despite some GI disorders being more prevalent in the elderly, there is no GI disease that is limited to this age group. While some changes associated with aging GI system are physiologic, others are pathological and particularly more prevalent among those above age 65 years. This article reviews the most important GI disorders in the elderly that clinicians encounter on a daily basis. We highlight age-related changes of the oral cavity, esophagus, stomach, small and large bowels, and the clinical implications of these changes. We review epidemiology and pathophysiology of common diseases, especially as they relate to clinical manifestation in elderly. Details regarding management of specific disease are discussed in detail if they significantly differ from the management for younger groups or if they are associated with significant challenges due to side effects or polypharmacy. Cancers of GI tract are not included in the scope of this article.

## 1. Introduction

The main characteristic of aging is progressive loss of physiological integrity which, in turn, leads to impaired function and increased vulnerability to death. This deterioration is the primary risk factor for the majority of diseases that affect humans including cancer, diabetes, cardiovascular disorders, and neurodegenerative diseases [1]. The elderly population is currently defined as people aged 65 years or above [2], yet increasing life expectancy may move this cut off upwards in years to come. The percent of elderly population varies by country with 7.8% in Turkey, 21.5% in Germany, and 14.5% in the United States (up from 10% in the 1970s) [2]. The predicted global population over the age of 80 years is expected to be 17% by 2050 [3]. Aging affects all functions of the gastrointestinal system (GIS): motility, enzyme and hormone secretion, digestion, and absorption. The GIS also plays an essential role in medication absorption and metabolism, and it is commonly affected by side effects. While there is no GI disease that is specific and limited to advanced age, some illnesses are more prevalent in this age group and may require different management. Hence, the focus of this review is to highlight the most common diseases that affect the elderly while emphasizing details of clinical presentation and management if they significantly differ from the younger population. Age-related pathophysiology and clinical implications in elderly are the main focus.

## 2. Oral Cavity

Changes in the oral cavity can be caused by local trauma (ill-fitting dental prosthesis, local radiotherapy), localized benign disease (aphthous stomatitis, oral candidiasis), benign systemic disease, potentially life-threatening conditions (vitamin deficiency, Sjogren's syndrome or Stevens-Johnson syndrome) or medication side effects (tricyclic antidepressants or antiparkinsonian drugs) [4, 5]. The most commonly reported disturbances among elderly include oral sensorial complaints (OSC), particularly dry mouth (xerostomia), taste disturbances (dysgeusia or ageusia), and burning mouth syndrome (BMS) [6]. These complaints may be explained by decrease in salivary secretion and quality of saliva associated with normal aging. Nagler and Hershkovic discovered that reduction in salivary function and altered composition are age-related [6, 7]. OSC are more prevalent in elderly than in those of age less than 65, with 50% of elderly reporting at least one of OSC. Among them, those using prescription drugs had higher prevalence of OSCs [6, 7].

**Xerostomia** may occur as a part of systemic disease like Sjogren's syndrome or as a medication side effect, with tricyclic antidepressants (TCA), atropine, and antiparkinsonian drugs being the most commonly implicated medications [4]. Additionally, damage to the salivary glands following radiation for head and neck cancers can lead to permanent xerostomia. When caused by medication, it is usually reversible upon the discontinuation of the offending drug [4]. Salivary supplements can be used to treat irreversible causes.

**Dysgeusia and ageusia** are also frequent among elderly and are most commonly caused by medication side effects. Common offenders include lithium, metronidazole, levodopa, glipizide, captopril, and clarithromycin. Zinc deficiency is another important cause of dysgeusia, especially in elderly who are frequently malnourished [4, 8]. Diseases of the peripheral nervous system (Bell's palsy) are more commonly associated with taste disturbances than are central nervous system disorders [8].

**Oropharyngeal dysphagia **is defined as a sensation of difficulty chewing food or initiation of swallowing [9], and results from changes affecting the complex neuromuscular mechanism that coordinates the tongue, pharynx, and upper esophageal sphincter (UES). The most common causes of oropharyngeal dysphagia are neuromuscular disorders such as stroke, multiple sclerosis, myasthenia gravis, dementia, Parkinson's disease, and muscular dystrophy. Pharyngeal cancers and strictures are local mechanical causes [10, 11]. Cough with swallowing, food sticking in the throat and nasal regurgitation are common signs of dysphagia and all increase risk for aspiration. Oropharyngeal dysphagia affects up to 13% of individuals above age of 65 and up to 50% of nursing home residents suffer from it [9, 12]. Elderly are usually not aware of their swallowing problems and might not seek help until complications such as aspiration pneumonia, malnutrition, or dehydration develop. Diagnosis is based on detailed history and by video fluoroscopic examination [5]. In many cases patients need artificial modes of feeding due to the irreversible nature of the underlying disease [10].  Table 1 summarize diseases of oral cavity in elderly.

## 3. Esophagus

Presbyesophagus is a term coined in 1964 [13] to describe the aging esophagus and changes that occur along this process. Over the last 40 years, with the development of more sophisticated diagnostic techniques, our understanding about age-related changes in the esophagus has evolved and current opinion is that presbyesophagus, in its original meaning, does not exist [14]. Esophageal problems in the elderly are more related to other co-morbidities rather than esophagus itself. The term presbyphagia is now used to describe characteristic physiologic changes in swallowing associated with the advanced age. While these changes are physiologic, they do increase the risk of dysphagia and aspiration in elderly, especially during acute illnesses and other stressors [14]. Studies have yielded conflicting results regarding esophageal motility in the elderly. Ferriolli et al. [15] demonstrated even healthy elderly patients exhibited significant changes in esophageal peristalsis and delayed esophageal emptying when compared to younger age groups. Two other studies [16, 17] found a significant decrease in peristaltic wave amplitudes as well as decreased LES and UES pressures. These studies demonstrated that the number of failed esophageal contractions and the duration of peristaltic contraction significantly increase in the elderly. Contrary to these studies, a retrospective study by Robson and Glick failed to demonstrate any significant difference in LES pressures and peristaltic duration and amplitude [18]. Finally, the most recent study by Besanko et al. [19] found only subtle changes in LES pressure and relaxation associated with aging. On the cellular level, these changes may be explained by loss of intrinsic enteric neurons which, in some studies have been shown to be more vulnerable to age-related degeneration and death than the other parts of the nervous systems. In particular cholinergic myenteric neurons seem to be more vulnerable than the other enteric neurons [20].

**Esophageal dysphagia **is most commonly manifested as the feeling of food being stuck in the chest. It can be caused by mechanical obstruction inside the esophagus itself (stricture, tumor) or compression from surrounding tissues (vascular compression or mediastinal masses). Neuromuscular causes include achalasia, scleroderma, or diffuse esophageal spasm. Finally, inflammatory and infectious causes can lead to esophageal dysphagia (eosinophilic esophagitis, candidiasis). Endoscopy is an essential part of the evaluation and can diagnose the majority of these conditions [9].

**Odynophagia** is painful swallowing. Infection and malignancy are the most common causes. Candida, herpes simplex virus, and cytomegalovirus are the most commonly found pathogens in infectious cases, especially in immunocompromised patients [21]. Several studies indicate elderly patients require more stimuli to perceive the disturbance and have a higher threshold for the sensation of pain [22, 23]. Lasch et al. [23] showed that elderly, nondiabetic patients required significantly higher mean balloon volumes to sense pain when compared to younger individuals. This may be explained by the previously mentioned age-related decrease of myenteric neurons. As a consequence, elderly patients frequently seek help late, when their disease is in advanced stage.

**Gastroesophageal reflux disease (GERD)** is a “condition that develops when the reflux of stomach contents causes troublesome symptoms and/or complications” unlike nonproblematic gastroesophageal reflux which is a physiological event that occurs during and after meals [24]. The prevalence of GERD among the elderly is about 23% and it was one of the most commonly found conditions among almost 20,000 nursing home residents in the study by Moore et al. [25]. While one would expect that prevalence of GERD increases with age due to multiple reasons (decreased saliva production and increased prevalence of motility disorders and hiatal hernia), studies demonstrated similar prevalence of GERD among older and younger age groups [26, 27]. However, severity of GERD and its associated complications (such as Barrett's esophagus, severe esophagitis, ulcerations and strictures) are more prevalent in the elderly [28, 29]. The most common symptom associated with GERD is heartburn. The frequency of severe heartburn seems to decline with age and the elderly might have atypical signs of GERD such as dysphagia or odynophagia [28]. This atypical presentation may relate to a decrease in esophageal pain perception and an increase in the prevalence of atrophic gastritis. Proton pump inhibitors (PPI) are the mainstay of treatment [29], yet the side effects associated with PPI therapy (increased risk of osteoporosis, development of Clostridium difficile colitis (CDC), interstitial nephritis, and community acquired pneumonia) should be kept in mind. While some authors voice concern regarding antireflux surgery in this age group [30], laparoscopic antireflux surgery should be considered in selected groups of patients. It appears to be a safe and effective treatment for GERD in elderly with low morbidity and mortality rates and excellent results [31].

**Barrett's columnar-lined esophagus (CLE) **is defined as a replacement of the normal distal esophageal squamous epithelium by metaplastic columnar epithelium, which is thought to be caused by prolonged GERD [32, 33]. The most feared complication of Barrett's esophagus is development of high grade dysplasia and adenocarcinoma. The incidence of adenocarcinoma in Barrett's esophagus has been estimated to be around 0.5% annually and slightly lower at 0.32% in patients without dysplasia at index endoscopy [33, 34]. A study by Gatenby et al. [33] found that risk of low grade dysplasia was independent of age at surveillance, yet they found that increasing age is an important risk factor for development of high-grade dysplasia and adenocarcinoma. The mean age at diagnosis of Barrett's esophagus was 61.6 and 67.3 years in males and females, respectively, and the mean life expectancy at diagnosis was 23.1 years in males, 20.7 years in females [35]. These findings indicate Barrett's esophagus is more prevalent among elderly. As previously mentioned (see chapter about GERD) older patients may be asymptomatic or have atypical symptoms, leading them to present late when complications may have already developed. Patients who are diagnosed with CLE may be offered surveillance in an attempt to detect dysplasia and potentially curable cancer. However, co-morbidities, life expectancy at the time of CLE diagnosis, and yearly risk of development of adenocarcinoma should be considered. A cross sectional study done by Myung S. Ko et al. found that one third of older men diagnosed with CLE had limited life expectancy [36]. They further argued that elderly patients are more likely to experience harms of surveillance than benefit from timely detection of dysplasia or early cancer and suggested that it should not be the routine strategy in this patient population.

**Medication induced esophagitis (pill esophagitis) **refers to the inflammation of the esophagus from harmful effects of medication due to their altered passage through esophagus and increased contact with esophageal mucosa. While it can occur at any age, it is more prevalent in elderly patients [37]. Polypharmacy, decrease in esophageal motility, and taking medication with insufficient amounts of water are factors most commonly associated with pill esophagitis in elderly [38]. Additionally, patients with cardiomegaly can develop esophageal compression from enlarged left atrium, placing them at increased risk for medication induced esophagitis. Abid et al. found female gender, presence of diabetes, and ischemic heart disease, in addition to advanced age, to be associated with pill esophagitis [38]. The most commonly implied causative agents include nonsteroidal anti-inflammatory drugs (NSAID), aspirin, doxycycline, bisphosphonates, ferrous sulfate, and captopril. The most common symptoms are chest pain (71.8%) and odynophagia (38.5%) [39]. Detailed history, availability of accurate medication list, and endoscopy are invaluable in establishing the diagnosis. Endoscopic findings show ulceration in more than 80% of cases, while kissing ulcers are present in up to 42% of patients [39]. The mainstay of treatment is withdrawal of the inciting medication and use of PPIs, leading to a favorable prognosis in most cases.  Table 2 summarize diseases of esophagus in elderly.

## 4. Stomach

Altered gastric microbiota, reduced mucosal protective mechanisms, decreased gastric blood flow, and consequently compromised repair mechanisms are the hallmarks of age-related gastric changes [40, 41]. These changes make older people more susceptible to the development of several diseases, such as gastric ulcer, atrophic gastritis, and peptic ulcer disease [41]. Additionally, elderly are more likely to experience medication related gastrointestinal side effects which, in turn, can decrease their medication adherence and further contribute to morbidity and mortality. The effect of age on gastric motility has been a subject of debate resulting in numerous studies reporting conflicting evidence. Using gamma camera technique, Madsen et al. [42] showed that gastric and small bowel motility were not reduced in healthy elderly subjects (mean age 81 year) when compared to control group (mean age 24 year). Additionally, they found that neither gender nor body mass index (BMI) influenced gastric and small bowel transit time. In contrast, a study by Shimamoto et al. [43] demonstrated significant decrease in postprandial gastric contractile force. They also found that this reduction was more pronounced among elderly who led less active lifestyles. Among the co-morbidities frequently encountered among older people, Parkinson's disease and diabetes mellitus seem to have the greatest impact on gastric emptying [44]. Loss of cholinergic enteral neurons seems the most plausible explanation for decreased motility in elderly. Although not yet seen in human studies, the current evidence for loss of these neurons is derived from animal models using rodents (rat, mouse and guinea pig). While there is so much more to discover about enteric neurodegeneration that occurs with aging, it seems reactive oxygen species (ROS) may play a central role and that, despite the absence of neurogenesis, the GIS has significant functional reserve as evidenced by relatively preserved function despite dramatic loss of enteric neurons [20].

**Chronic atrophic gastritis (CAG) **is more prevalent in the elderly and is associated with H. pylori infection [45]. The hallmark of disease is the partial loss of glands in the gastric mucosa leading to hypochlorhydria or achlorhydria. The prevalence of CAG is higher in elderly and there are significant geographical variations showing elderly from China and Japan are particularly affected with a prevalence of up to 50% in those above age 60 years [45, 46]. While advanced age was thought to be independently related to chronic atrophic gastritis in the past, current data support the theory that atrophic changes of the gastric mucosa are associated with H. pylori infection rather than the age itself [47]. Gastritis activity can be decreased by eradication of H. pylori [47, 48]. In their study, Kokkola et al. [49] demonstrated that elderly patients who successfully eradicated infection had significantly lower mean histological scores of inflammation, atrophy, and intestinal metaplasia. Decrease in acid secretion as a consequence of chronic atrophic gastritis leads to two problems that are particularly prominent in the elderly population: small intestinal bacterial overgrowth (SIBO) and malabsorption [50, 51]. A study by Parlesak et al. examined prevalence of SIBO using hydrogen breath test and found the prevalence in elderly was 15.6% compared to 5.9% found in younger age groups [51]. Several studies examined the association between CAG and other co-morbidities in elderly. A cross sectional study by Hye Won et al. found significant association between presence of CAG and development of osteoporosis in elderly females in Korea [52]. They explained their findings were a result of decreased calcium absorption secondary to hypochlorhydria or achlorhydria leading to decreased bone mineral density. Another cross sectional study [53] found a positive association between autoimmune thyroid disease and CAG. The authors suggested that autoimmune thyroid disease and atrophic body gastritis occur in a closely linked fashion, particularly in females with positive parietal cell antibodies. Although data are still somewhat conflicting, recent studies suggest that eradicating H. pylori infection in elderly patients may prevent progression from CAG to intestinal metaplasia and gastric cancer (GC) [54]. Emerging knowledge about the changes in human gastric microbiota associated with aging has shed new light on its association with gastric cancer. Pearson and co-authors [41] showed that gastric microbiota varies for patients with autoimmune and H. Pylori associated atrophic gastritis, with former expressing higher bacterial diversity and abundance. Both conditions are associated with reduction in gastric acid secretion and development of gastric cancer (particularly neuroendocrine and gastric adenocarcinoma), while people treated with PPIs maintain normal gastric microbiota diversity in spite of hypochlorhydria [41].

**H. pylori infection **prevalence in developing countries is the highest among the children, while in developed countries is higher with increasing age [55]. This marked difference is most likely due to a cohort effect of the earlier generation exposed to poor sanitation [47] which is a known risk factor for contracting the infection. On the global level, 50% of people are colonized by H. pylori [56] and the prevalence varies from 7% to 87% [55]. Apart from being associated with peptic ulcer disease, H. pylori is identified as a type I carcinogen associated with gastric adenocarcinoma and gastric non-Hodgkin's lymphoma [57, 58]. Severity of upper gastrointestinal symptoms secondary to H. pylori infection seems to be higher in the elderly [47]. Several extra intestinal disorders have been associated with H. pylori seropositivity. The most notable of these for older adults include cardiovascular diseases, osteoporosis, and neurocognitive impairment. Several studies have established association between H. pylori infection and cardiovascular diseases [59, 60], yet a few other studies failed to find any association [61, 62]. Chronic inflammation secondary to ongoing H. pylori infection may play a role in developing or worsening Alzheimer dementia [63–65] and vascular dementia [66]. The association between compromised bone mineral density and H. pylori infection has been supported by recent investigations [52, 67]. Despite evidence associating H. Pylori infection with extra intestinal diseases (dermatological, neurologic, cardiovascular and hematologic), further studies are needed to confirm this relationship [68]. Current evidence indicates H. Pylori's strongest associations are with iron deficiency anemia (IDA) and immune thrombocytopenic purpura (ITP)[68].

**Peptic ulcer disease (PUD)** encompasses both gastric and duodenal peptic injury that leads to a break in gastric or duodenal mucosa and, more rarely, in the esophagus or Meckel's diverticulum [69]. The majority of ulcers in elderly is caused by H. pylori infection or is associated with the use of NSAIDs/aspirin [69, 70]. Unlike in general population, the incidence and mortality from PUD in elderly remains very high [71]. Gastric ulcers in elderly are usually larger and tend to occur higher in the stomach on the lesser curve [72]. The main factors contributing to this are a higher prevalence of H. pylori infection among elderly, increased NSAIDs/aspirin use, and polypharmacy including medications associated with increased risk of PUD (anticoagulants, selective serotonin reuptake inhibitors (SSRIs), and oral steroids). The physiologic changes associated with old age that contribute to this include reduced blood flow through GI system and decreased secretion of key components of gastrointestinal protective mechanisms such as bicarbonates, mucin, and prostaglandins [71]. Gastric acid secretion is unaffected in healthy elderly [73], but H. pylori infection associated changes in gastric microbiota and atrophic gastritis both lead to hypochlorhydria and a decline in gastric acid secretion in older adults [5, 41].

Clinical manifestation of PUD in elderly is often atypical. In their prospective study, Hilton et al. demonstrated that only 30% of elderly patients with endoscopy proven PUD had typical epigastric pain [74]. Additionally, elderly patients with acute gastric or duodenal ulcer perforation might not exhibit classic signs of chemical peritonitis [75], which is partially due to hypochlorhydria observed in this age group. While medical treatment of PUD in elderly is not significantly different than in younger patients, medication side effects and interactions must be kept in mind. Many elderly have a strong concomitant indication for antiplatelet or anticoagulant agents, making exploring both individualized risks and benefits of continuing those along with H2 blockers or proton pump inhibitors (PPIs) crucial. There is emerging evidence that PPIs are associated with community acquired pneumonia (CAP), Clostridium difficile colitis (CDC) and osteoporosis, all of which tend to be more severe in elderly. Significant correlation between hip fractures and the use of PPIs in elderly has also been reported, leading to concerns that duration of treatment should depend on indication and should not be universally lifelong [76]. Elderly with bleeding PUD who underwent open surgical repair had significantly higher 30 day morbidity and mortality then younger counterparts [77]. A retrospective study by Fezzi at all [78] analyzed outcomes for 98 patients admitted for bleeding peptic ulcer and found that 66% achieved hemostasis endoscopically. They found repeated endoscopic treatment was not associated with increased mortality and recommended repeated endoscopy in elderly with elevated surgical risk. Trans-catheter arterial embolization was another option to consider in this group based on the same study.  Table 3 summarize diseases of stomach in elderly.

## 5. Small Intestine

The hormonal secretion and absorptive function of the small intestine in older adults is not significantly different in comparison to younger counterparts [5]. Minimal changes in small bowel motility as well as reduced mucosal immunity are seen, but they are clinically insignificant in the absence of co-existing illness. While some animal studies demonstrate age-related changes in small intestinal morphology such as increase in villous width and height and reduction in mucosal surface area, human studies did not find such changes [79]. There are few human studies on this topic and while some morphologic changes may occur they usually are not severe enough to be a cause of malabsorption [80]. The studies done by Ciccocioppo and Corazza [81, 82] showed that increase in proliferation and differentiation rates of enterocytes maintains unchanged intestinal architecture with increased expression of proliferating cell nuclear antigen in elderly compared to younger age groups.

**Celiac disease (CD)** is a chronic, systemic autoimmune disease that affects genetically predisposed individuals secondary to exposure to dietary gluten protein [83]. The classic form of CD manifests as a malabsorption syndrome associated with chronic diarrhea, mineral deficiencies, failure to thrive, and weight loss. Many patients have only extra intestinal manifestations or even no symptoms at all. These forms are termed atypical and silent celiac disease, respectively [84]. In patients who suffer from celiac disease, tissue transglutaminase (TTG) binds to gliadin in the gut level. Antigen presenting cells which express HLADQ2 and HLADQ8 present this gliadin-tissue transglutaminase complex to T cells which then stimulate B cells to produce antibodies against both gliadin and TTG [85]. Typical symptoms of celiac disease are frequently absent in elderly which leads to diagnostic delay, increasing morbidity in this age group [86]. Evidence suggests increasing incidence of celiac disease in older adults in North America as seen in Olmsted County, Minnesota, USA, with 15.1 new cases per 100.000 in 2000-2001 compared to zero in 1950-1559 [87]. While some of these cases might be diagnosed in elderly for the first time due to diagnostic delay, there is evidence that celiac disease can truly occur for the first time in elderly in spite of long tolerance to gluten in the past [88]. As is typical in many autoimmune diseases, it is typically more frequent in females, yet we see that after age 65 incidence in women starts decreasing while it gradually increases in men [87]. It is unclear why gastrointestinal symptoms are less prominent in elderly patients with celiac disease, and the first sign of disease can be micronutrient deficiency [89]. Iron-deficiency anemia is present in up to 80% of elderly patients with celiac disease [90]. When abdominal symptoms are present in these patients, they are usually vague and nonspecific, manifesting as bloating, increased flatulence, and abdominal discomfort [83, 89].

Calcium and vitamin D deficiency are frequently found in celiac disease, which leads to metabolic bone disease and increased risk of fractures, especially with falls occurring at an older age. Liver involvement in celiac disease, seen in up to 20% of patients, is termed celiac hepatitis and usually presents as hypoalbuminemia, ascites, and abnormal liver function tests [91, 92]. Interestingly, celiac hepatitis is not auto-immune mediated disease unlike cholestatic liver diseases like primary biliary cholangitis, primary sclerosing cholangitis, and auto-immune hepatitis, which are also more common in people who have celiac disease [85]. Unlike celiac hepatitis, which usually resolves with gluten free diet, cholestatic liver disorders respond less favorably on gluten cessation.

Neurologic manifestation of celiac disease, particularly dementia, ataxia, and neuropathy, are especially difficult to diagnose in older adults due to the broad differential diagnosis and unfamiliarity amongst providers that celiac disease can present with neurological manifestations. This age group will frequently be misdiagnosed with Alzheimer or vascular dementia or gait instability due to age and deconditioning when, in fact, they may have atypical celiac disease. Apart from celiac disease, malabsorption syndrome in elderly might also be due to small bowel ischemia, bacterial overgrowth, and exocrine pancreatic insufficiency which makes diagnosis of celiac disease in these groups of patient with steatorrhea even more challenging.

While the diagnostic algorithm of celiac disease in elderly is the same as in younger groups, management can be particularly challenging since these patients can find complying with a strict gluten free diet more difficult. Some of this difficulty connects to residing in assisted living facilities, inability to precisely read ingredients while shopping, and finding lifelong habits difficult to break [85].

**Mesenteric ischemia** results from occlusion of celiac axis arterial branches and can be either acute or chronic. Its pathophysiology is related to either arterial occlusion by embolus (superior mesenteric artery affected in more than 50% of cases), gradual development of thrombosis secondary to advanced atherosclerosis, or hypo-perfusion secondary to hypovolemia and hypotension. Historically, embolic pathophysiology was a more common cause of acute mesenteric ischemia (AMI), yet more recent trends indicate atherosclerotic etiology is now more prevalent [93]. This may be due to increased life expectancy and population of elderly who more commonly have severe atherosclerotic changes as well as increased use of anticoagulation which will decrease embolic phenomena. Elderly are disproportionally affected by nonocclusive mesenteric ischemia related to states of hypo-perfusion such as during cardiopulmonary bypass surgeries, dehydration, hypovolemia, sepsis, and states of shock. Additionally, splanchnic blood flow decreases with age, making elderly more vulnerable to this type of hemodynamic injury. Chronic mesenteric ischemia (CMI) in elderly classically presents as post prandial angina leading to decrease in food intake and subsequent weight loss. Abdominal pain, which is a hallmark of presentation of acute mesenteric ischemia, in elderly is frequently absent or minimal and vague [94]. In contrast, CMI frequently presents with tachypnea, confusion, vomiting, and diarrhea. The onset of symptoms is usually insidious and when combined with ambiguity of symptoms makes establishing the correct diagnosis extremely difficult. A textbook definition of poorly localized abdominal pain out of proportion to the physical findings applies only to acute embolic mesenteric occlusion of a previously patent artery and is rarely seen nowadays.

Acute mesenteric ischemia is a more common abdominal emergency than ruptured abdominal aneurysm [95] and, in people older than 75, is a more common cause of acute abdomen than appendicitis. Elderly patients who have symptomatic chronic mesenteric ischemia are at very high risk of developing acute on chronic mesenteric ischemia. In fact, one study demonstrated that up to 80% of patients who developed acute on chronic mesenteric ischemia had been evaluated for similar complaints within 6 months before acute ischemia developed [96]. Diagnosis of AMI should be based on a combination of clinical suspicion (look for risk factors like peripheral arterial disease, atrial fibrillation, prior myocardial infarct, or stroke), laboratory findings (leukocytosis, elevated lactic acid, and metabolic acidosis) and radiological findings (thrombus in the artery, increased bowel enhancement, pneumatosis, etc.). Sensitivity of CT is variable, especially early in the disease, and can be increased in the setting of high clinical suspicion. In study by Karkkainen only 50% of patients with documented bowel ischemia had CT findings of thrombus in the artery [93]. Studies by Schermerhon and Arthus showed that endovascular therapy is associated with lower mortality when compared to open revascularization (16% vs 28%) [97, 98] and is preferable in elderly who usually have a plethora of other co-morbidities making them poor candidates for open surgery.

**Small bowel bleeding** (SBB) makes up only 5% of GI bleeds, yet it is the most common cause of obscure GI bleeding. The small bowel is defined as the region between the ligament of Treitz and the ileocecal valve. It is called “the dark continent of the GI tract” due to its excess mobility, long length, and relative inaccessibility to endoscopy. Unlike for young adults in whom intestinal tumors and Crohn's disease are the most common causes of small bowel bleeding, vascular anomalies, small intestinal ulcers, and NSAID enteropathy are the most common etiologies in elderly populations [99]. A study by Zhang et al. [100] found that 54% of small bowel bleeding in elderly patients was due to angiodysplasia.

**Angiodysplasias** are acquired lesions associated with aging. They are characterized by the presence of a cluster of dilated, torturous, thin-walled vessels involving small capillaries, veins, and arteries [101]. The increase in incidence of angiodysplasia with age is thought to be due to changes in the composition and structure of extracellular matrix in the wall of the small intestine. They are associated with Heyde's syndrome, a well described clinical syndrome of bleeding from angiodysplasia in patients with aortic stenosis, which incidence increases with increasing age [102]. Another condition associated with increased frequency of GI angiodysplasia is chronic renal failure. Study by Karagiannis et al. found that 47% of patients with chronic renal failure had small bowel angiodysplasia compared to 17.6% in people with normal renal function [103]. Apart from patients with aortic stenosis and chronic renal failure, newly recognized risk factors associated with presence of small bowel angiodysplasia include female gender, chronic respiratory condition, VTE, and use of warfarin [104].

**Small bowel ulcers** are another common cause of SBB in elderly. Interestingly, one study from India found small bowel ulcers to be a more common cause of obscure GI bleed than angiodysplasia [105]. While Crohn's disease ulcers are more common in young adults, NSAID-associated small bowel ulcers are the most common cause in elderly. The prevalence of small bowel ulcers increases with age. When compared to young adults (below 40) who have a 7.27% incidence of small bowel ulcers, elderly (above 65) have an almost double incidence of 13.04% [100].

Finally, elderly patients who are more likely to have abdominal aortic aneurysm and undergo surgical or endoscopic repair are at high risk for development of aorto-enteric fistulas which are another important but uncommon cause of SBB in elderly [106]. Unlike bleeding from angiodysplasia that is usually slow and recurrent, this type of bleeding is usually massive and life-threatening with high mortality in elderly.

A study by Compagna et al. found that most elderly patients treated with either argon plasma coagulation(APC) or bipolar electrocoagulation(BEC) achieved good hemostasis after the first cycle. They also found that recurrence of bleed was lower in patients who were treated with APC (10%) in comparison to those treated with BEC (20%). Overall they concluded that elderly showed good response to endoscopic treatment [107].

**Small intestinal bacterial overgrowth (SIBO)** implies excessive presence of bacteria, above 10^5^-10^6^ organism/mL in small bowel aspirate [108]. Common in elderly; it is associated with chronic diarrhea, malabsorption, weight loss, and secondary nutritional deficiencies. Prevalence of SIBO in the healthy population is significantly higher among elderly when compared to younger adults, 15.6% vs 5.9% [51]. The prevalence of SIBO in healthy elderly adults in UK was found to be 14.5% [109]. Interestingly a study from Japan that included healthy elderly (mean age 74 years) found no patients with SIBO [110]. It seems that exact prevalence of SIBO varies between the countries and geographic location. It is higher among elderly, especially those hospitalized or in long term care facilities. Several factors contribute to the development of SIBO such as achlorhydria, small intestinal dysmotility, increased transit time, alteration in gut immune function, and anatomical abnormalities (bowel resection, presence of anastomosis, and fistulas). Among these, the two primary factors associated with risk of SIBO are achlorhydria and small bowel dysmotility, both of which are more common in elderly [111]. Achlorhydria, as previously mentioned, is associated with use of medications such as PPIs and H2 blockers as well as the presence of H.pylori infection. Decrease in gastric acid production allows bacteria to pass through the stomach and overpopulate the small intestine. The risk of SIBO appears to be higher in patients treated with PPIs than H2 blockers. Gastroparesis with impaired gastric transit time and delayed gastric emptying can increase risk of SIBO due to food and bacterial stasis in the upper gastrointestinal tract. Small bowel motility disorders predispose patients to SIBO because bacteria are not effectively swept distally to colon. Patients with diabetes, portal hypertension, chronic renal failure, scleroderma, and polymyositis have higher risk of development of SIBO in comparison to healthy patients [112, 113].

Motility of the small intestine is not affected by age itself. Rather than age itself, slower motility in elderly is associated with medications, polypharmacy, and presence of concomitant diseases more frequently seen in this population such as autonomic neuropathy from long standing diabetes. Clinical manifestation of SIBO can typically include nausea, vomiting, and diarrhea [108]. In the elderly, these may be not as prominent and manifestations of the disease can be more subtle and include nonspecific abdominal distention, bloating, and poorly localized discomfort may mimic other diseases such as irritable bowel syndrome, celiac disease, lactose intolerance, fructose intolerance, or inflammatory bowel disease. Symptoms of SIBO complications due to malabsorption, such as vitamin and mineral deficiencies, might be the first sign in elderly. Unlike vitamin B12 deficiency that is commonly seen in SIBO due to competitive uptake of vitamin B12 by bacteria, vitamin K and folic acid are usually normal in patients with SIBO since they are produced by gut bacteria. Diagnosis of SIBO is similar to young adults and includes small bowel content aspiration and, more commonly, glucose or lactulose breath tests [108]. Treatment includes dietary changes emphasizing low carbohydrate diet, increasing GI motility by use of prokinetic agents, and reduction of bacterial overgrowth using antibiotics. Due to frequent presence of polypharmacy and medication interactions, use of prokinetic agents is especially challenging in elderly populations. Additionally, use of antibiotics is associated with development of CDC which carries high morbidity and mortality in this age group. Hence, the risk and benefits of using antibiotics and prokinetic agents should be weighed on and individual basis in elderly.  Table 4 summarize small intestine diseases in elderly.

## 6. Large Intestine

Several studies tried to address whether the process of aging itself negatively impacts gastrointestinal motility and increases colon transit time. The results from these studies yielded conflicting results and at this point it remains unclear whether or not age itself is a risk factor for development of motility disorders of the large intestine. Metcalf et al. found that aging itself doesn't influence colon transit time, while Madsen and Graf found the opposite—increased colon transit time in people age 80 and above [42, 114]. Despite these discordant observations, most experts agree that constipation is not a physiologic consequence of normal aging. In fact, most healthy older people have normal bowel function. If some degree of motility impairment due to age is present, it might be related to decreased ability of enteric smooth muscles to contract and relax or to changes in enteric nervous system and reduction in the concentration of neurotransmitters [50, 115]. Hanani et al. documented observations that the myenteric plexus of the human colon undergoes changes with aging that manifest as increases in cavities of myenteric neurons [116]. On the other hand, Bernard and colleagues found that neuronal loss in the myenteric plexus was specific to cholinergic neurons while nitrinergic neurons were spared [117]. Decreasing synthesis of neurotransmitters is another theory that attempted to explain potential decrease in colon transit time associated with aging and Takahashi et al. documented a significant decrease in nitric oxide synthase (NOS)-immunoreactive cells as well as NOS synthesis in colonic neurons [118]. In contrast to the above findings, a study by Southwell et al. found that healthy elderly people do not have significant reduction in numbers of submucosal myenteric neurons [119]. While we are awaiting further studies to clarify these conflicting results, it seems that age-related changes in the colon are clinically insignificant in the absence of other conditions that predispose elderly to decreased colon transit time and constipation such as anticholinergic medications, decrease in mobility, dietary changes, and comorbid medical conditions.

Another important change that occurs along the process of aging is the change in human intestinal microbiota [120]. There is a high degree of variability between infant microbiota (dominated by* Bifidobacterium*) and microbiota of an adult person (Bacteroidetes and* Firmicutes* dominate). Age-related changes in human microbiota have been associated with inflammatory bowel diseases (Crohn's disease and ulcerative colitis), irritable bowel syndrome, and metabolic disorders (diabetes mellitus types 1 and 2 and obesity). Two major* phyla* of human microbiota are* Firmicutes* (gram positive bacteria) and* Bacteroidetes *(gram negative bacteria) [120]. Immune homeostasis by intestinal microbiota is maintained by the equilibrium between these two major* phyla*. Age-related alteration in this balance may lead to activation of dendritic cells within the lamina propria of the intestine which, in turn, starts the cascade of events leading to release of pro-inflammatory cytokines, mainly interleukins 6 and 17. These alterations further lead to decreased secretion of mucus and *α*-defensins by intestinal epithelial cells (IEC), which then allows entry of pathogens into mucosal layers, finally resulting in generation of low grade inflammation, “inflamm-ageing” [121]. Short chain fatty acids (SCFA) produced by healthy microbiota have anti-inflammatory and antineoplastic properties. In elderly, age-related alteration of microbiota leads to decrease in production of SCFA which may promote inflammation as well as decrease function of IECs [122].

**Constipation **is defined by most clinicians as a decrease in defecation frequency to three or fewer per week and Rome criteria are the most frequently used consensus definitions clinically and for research [123]. The estimated prevalence of constipation in the general population is reported to be anywhere from 2%-28% [124]. In elderly, this number goes up to 40% [125] and up to 50% of elderly nursing home residents [126]. Constipation is also more common in females, African Americans, and persons from lower socioeconomic status [127]. Increase in prevalence of constipation in elderly is not related to decrease in colon transition time as much as it is to decreased mobility, cognitive impairment, comorbid medical problems, polypharmacy (especially opioid and anticholinergic medication use), and dietary changes. Elderly patients usually associate constipation with straining rather than decreased frequency of bowel movements. Primary constipation can be divided in three groups: (1) normal transit constipation; (2) slow transit constipation and (3) anorectal dysfunction. The most common among these is normal transit constipation which is also called functional constipation. Anorectal constipation might be secondary to age-related changes in anorectal physiology including increased rectal compliance, impaired rectal sensation, and impaired defecation [128]. In this type of constipation, inefficient coordination between pelvic musculature and evacuation mechanism often occurs. Physiologic changes that contribute to constipation in elderly have less to do with increased colonic transit time and more with anorectal function changes. There is a significant overlap between normal transit constipation and irritable bowel syndrome (IBS), with the main clinical difference between these two being predominance of abdominal pain and discomfort among elderly with IBS [129]. Secondary constipation is either due to medication side effect (calcium channel blockers, opioids, NSAIDs, iron pills, antacids) or associated with countless other diseases including endocrine (hypothyroidism, hypercalcemia), neurologic (Parkinson's disease, neurogenic bowel due to spinal cord injury, stroke), rheumatologic (scleroderma, amyloidosis) and psychological (somatization,depression). A major and most feared complication of constipation in elderly is stool impaction, which can lead to stercoral ulcerations and colonic perforation which carry high mortalities in elderly populations. Fecal impaction refers to accumulation of hardened feces in the rectum or colon which causes diminished rectal sensation and subsequent fecal incontinence [128]. Symptoms of fecal impactions are constipation associated with abdominal pain, urinary symptoms, respiratory distress, and even fever in severe cases. Liquid stool from the proximal colon can bypass the impacted stool causing paradoxical diarrhea, so the presence of diarrhea alone cannot exclude fecal impaction.

**Diverticular disease (DD)** is a term used to describe two conditions: diverticulosis and diverticulitis. Combined, they are the most common disease affecting the large bowel in the Western world, with the highest rates in the Unites States and Europe showing no gender predilection [130]. The prevalence of DD has increased over the past decades throughout the world and while it can affect any adult patient, the prevalence increases with age [131]. DD is uncommon in those under age of 40 with estimated prevalence of 5% and quite common by age 80 with an approximate prevalence of 70% in the US [130–132]. Diverticulosis is an acquired condition referring to presence of diverticula—sac like outpouchings of mucosa and submucosa of colonic wall. They are believed to develop due to increased intraluminal colonic pressure at the points of least resistance in the muscular wall where vasa recta insert. While it is primarily a left colon disease in people from Western societies largely affecting the sigmoid and descending colon in 90%, in Asians it mostly often affects the right side with the cecum and ascending colon being affected in 55%-71% [133, 134]. The importance of a genetic component in the occurrence and location of DD is exemplified in the fact that in the Japanese Hawaiian community, the dominant site of DD occurrence has remained on the right side as it has in the indigenous Japanese population [135].

Diverticulitis is considered the most common complication of diverticulosis and refers to inflammation of diverticula that can vary in severity and is associated with its own complications such as bleeding, micro-perforation, abscess formation, and/or secondary bacterial peritonitis. Diverticular colitis represents intense inflammation of colonic mucosa in and around areas of multiple diverticula manifested as abdominal pain, change in bowel habits, and hematochezia [136].

A growing body of evidence is shifting the paradigm of diverticular disease from being an acute surgical disease to a chronic bowel disorder composed of recurrent flares with intervals in between filled with either relatively asymptomatic times or vague and recurrent abdominal discomfort [136]. These relatively milder symptoms in periods between the flares might mimic other gastrointestinal disorders, such as irritable bowel syndrome (IBS). It is unclear whether ongoing mild symptoms after an episode of diverticulitis flare result from ongoing low grade inflammation that continues after acute symptoms resolve or from previously unrecognized IBS. It can be possible that an episode of diverticulitis exacerbates or/and triggers IBS similar to already described post infectious IBS. Hence, DD presents with the spectrum from asymptomatic presence of diverticulosis to symptomatic uncomplicated diverticular disease to, finally, complicated disease [130]. Pathophysiology of DD is linked to age-related changes in the connective tissue of the colonic wall which include an increase in collagen crosslinking and increased elastin content both leading to increased colonic wall rigidity [137]. Additionally, it incorporates a complex interplay between colon microbiota, inflammation, visceral hypersensitivity, and colonic motility [136].

The natural history of diverticulitis is poorly understood and research on this topic has been lacking. Traditionally it is believed that among those with diverticulosis, about 25% will go on to develop the most common complication—diverticulitis [138]. More recent studies, however, described the incidence of diverticulitis to be as low as 1-2% among those with diverticulosis [139, 140]. A study by Strate et al. found an overall incidence of less than 5% contrary to previously held dogma that 25% of patients with diverticulosis will develop diverticulitis in their lifetime [136]. When they applied the stricter criteria for the diagnosis of inflammation, including CT documented disease, this incidence further decreased to 1%. Interestingly they noted that younger patients, in comparison to elderly, had significantly higher incidence of diverticulitis per year suggesting that younger age may be a risk factor for development of diverticulitis. Additionally, diverticulitis developing in younger patients more often becomes complicated [130, 136]. Hogan et al. [141], in their series of 930 patients with diverticulosis but without diverticulitis, found that 75% had evidence of inflammation in and around diverticula. It led some authors to propose that diverticulosis might be either a milder form of inflammatory bowel disease (IBD) or tightly connected to it [142]. It further led to exploring of use of mesalamine in the treatment and prevention of diverticulosis and diverticulitis, respectively. Connecting to the idea that imbalance in colonic microbiota may be responsible for the development of diverticulosis and its complications, there are ongoing studies investigating use of rifaximine, alone or in combination with mesalamine, for treatment and prevention of DD [143, 144].

While some medications commonly used by elderly (such as NSAIDS, steroids and opioids) are linked with an increase in the risk for DD [145, 146], others like calcium channel blockers and statins showed protective effect [147]. Hence, it would be reasonable to use calcium channel blockers as first line antihypertensive in patients with concomitant hypertension and DD in the absence of stronger indications for other comorbidities (for example beta blockers in known coronary artery disease or angiotensin converting enzyme blockers in patients with diabetes and hypertension). Elderly people with diverticulosis are at increased risk of ischemic colitis which, unlike diverticulosis which usually presents as painless hematochezia, is usually associated with abdominal pain and elevation in lactic acid. It appears that elderly patients with atherosclerosis are more prone to develop diverticular bleeding then controls without atherosclerotic diseases [132].

Studies from UK demonstrated that patients with DD have lower health related quality of life [148]. Even patients with uncomplicated diverticulosis have lower quality of life when compared with unaffected age and sex matched controls [149].

Following resolution of an episode of diverticulitis, it has been a standard of care to have a colonoscopy to rule out underlying malignancy mimicking diverticulitis. A recent retrospective study and systematic review, however, showed that incidence of malignancy in these cases is truly low [150]. Hence, follow up colonoscopy might need to be limited to those with persistent symptoms, alarm findings, or suspicious CT findings.

**Irritable bowel syndrome (IBS)** is common functional gastrointestinal disorder. It manifests with abdominal pain and alteration in bowel movements in the absence of any organic pathology [151]. Symptom-based criteria known as Rome criteria are the most widely accepted method of diagnosing IBS since there is no pathognomonic result or finding [152]. Depending on bowel patterns, IBS can be diarrhea predominant, constipation predominant or IBS with mixed bowel habits. The prevalence of IBS seems to be similar in elderly and younger people [153], yet the incidence of IBS is highest in adolescence and is rarely diagnosed for the first time after age 65. While IBS symptoms are not significantly different across age groups, elderly are more likely to have organic gastrointestinal disease, hence, a careful diagnostic approach should be used in this particular group. Clinicians who provide care for elderly frequently encounter patients who have multiple GI complains, take various medications that might affect GI system, and have a plethora of other co-morbidities. Taking all these factors into account, it is difficult to confidently diagnose IBS in such circumstances. In fact, a prospective study, done on sample of 230 patients visiting elderly care clinic in the UK found that a striking minority (only one patient) with IBS was actually diagnosed with the disease, despite 22% having symptoms suggestive of IBS [154]. It seems that clinicians are more likely to attribute GI symptoms in elderly to an organic or medication-related etiology than to a functional disorder. Traditional approaches that argue for minimal investigation once IBS is suspected are plausible in younger people without alarm features, especially if accompanied with psychological disorders of anxiety, depression or somatization. In elderly, however, who are more likely to have alarm features, this approach is not realistic and IBS should be a diagnosis of exclusion in the appropriate clinical setting. Rectal bleeding is considered an alarm feature but is frequently found in patients with IBS of any age [155], whereas weight loss and poor appetite are “red flag symptoms” that are commonly found in elderly irrespective of the presence of IBS. In elderly male patients, chronic prostatitis can mimic IBS and present as intermittent diarrhea, passage of mucus, abdominal pain, and difficulties in evacuation. Pathophysiology of IBS is not clear and multiple theories such as altered gut motility, visceral hypersensitivity, psychosocial theory, altered gut microbiota, and post infectious have been proposed [156]. These are similar to theories about pathogenesis of diverticulitis. One recent theory argues that gut motility affected by 5-HT (serotonin) concentration might be responsible for IBS [157, 158]. In fact, we know that serotonin is a mediator in initiating colonic motility and increased serotonin concentrations have been found in diarrhea predominant IBS while patients with constipation predominant IBS were more likely to have lower concentrations of serotonin. IBS treatment is the same across different age groups, yet medication side effects and polypharmacy can lead to cautious use in older adults. Patients treated with selective serotonin reuptake inhibitors had significantly higher response and improvement in their IBS symptoms when compared to the group treated with tricyclic antidepressants (TCA) [159]. TCA and antispasmodic medications can exacerbate urinary retention, closed angle glaucoma, and cognitive impairment in elderly secondary to their anticholinergic properties. Among nonpharmacological therapies, CBT seems to be associated with the best results.

**Clostridium difficile colitis (CDC)** is a major cause of GI infection worldwide and occurs in adults aged 65 and above in up to 80% of cases [160]. Elderly populations are particularly vulnerable to this infection and suffer higher morbidity and mortality when compared to younger counterparts [161]. CDC occurs due to dysregulation of gut microbiota and most commonly is due to use of antimicrobials [162]. The virulence factors of C. difficile are toxin A and toxin B, which exhibit their pathologic effect by damaging colon epithelium and generating an acute neutrophil predominant inflammatory response resulting in the hallmark macroscopic manifestation of CDC—formation of pseudomembranes.

Clinical manifestation varies and includes mild diarrhea, moderate disease accompanied by dehydration and acute kidney injury, and severe life threatening disease with toxic megacolon accompanied by sepsis and septic shock [163]. The most severe forms of the disease disproportionally affect elderly people, nursing home residents, and malnourished patients [137]. The incidence of CDC has dramatically increased over the last few years, with rates tripling in the Unites States and Canada [164]. CDC hospitalization rates are significantly higher in those of age 65 and above (by fourfold) and especially those older than 85 (by tenfold) when compared to groups younger than 65 [165]. Not only did the incidence of CDC increase, but mortality did as well, from 5.7 deaths per million in 1999 to 23.7 in 2004, despite significant advancement in our knowledge about preventive measures, early recognition of infection, and appropriate treatment [166]. This increase in mortality is mostly driven by the emergence of more virulent strain of bacterium called NAP-1 (North American pulsed –field 1/PCR ribosome 025] [167]. Increasing age and the use of fluoroquinolone are identified to be risk factors for infection with the NAP-1 strain [168]. A prospective cohort study from Canada identified age above 65, antibiotic exposure, and use of proton pump inhibitors to be significantly associated with health care associated CDC [169]. In addition to more severe forms of disease and risk for health care associated infection, age is also a risk factor for recurrent disease [170]. CDC recurrence can be predicted by the Hu prediction tool [171]. Three clinical factors emerged to be the most important for prediction of CDC recurrence: age above 65, fulminant or severe underlying co-morbidities, and additional antimicrobial use following initial treatment for CDC. Aging is associated with alteration in important physiologic barriers to infection including immunosenescence, which is a complex age-related change in the immune system that makes elderly more susceptible to infections [172]. Immunoscenescence is directly related to decreases in T and B cell numbers and the decline in their immunologic function [173]. A particularly important step in the pathogenesis of severe CDC in elderly is a decrease in diversity of GI microbiome associated with aging [174], which contributes to dysregulation of gut microbiota and predisposes older individuals to development of CDC. Decreased functional status is also an independent risk factor for poor outcome in older adults [137].

Treatment of CDC includes discontinuation of the precipitating antimicrobial whenever possible, IV hydration, and initiation of anti C. difficile antibiotics. While initial studies demonstrated equal effectiveness of metronidazole and vancomycin, several new studies showed superiority of vancomycin over metronidazole in elderly people with severe disease [175–177]. Increased treatment failure in the older adults was associated with metronidazole therapy in a systematic review by Vardakas [175]. These and a few other observations lead the Infectious Disease Society of America (IDSA) to recommend Vancomycin as initial empiric therapy in the newest guideline from 2018 [178]. Metronidazole is systemically absorbed and may cause side effects such as nausea, dysgeusia, seizures, peripheral neuropathy or encephalopathy. Vancomycin and fidaxomicin, on the other hand, are not systemically reabsorbed leading to a better side effect profile, which is especially important in the elderly who are frequently on multiple other medications. Fecal microbiota transplant (FMT) has emerged as effective treatment for recurrent C. difficile infection who failed multiple antibiotic treatments. FMT, similar to other treatment options, is also associated with higher overall recurrence rate among older groups (9.3%) compared to younger populations (4.6%) [179]. Additionally, due to age-related changes in gut microbiota and diversity of microbiome, elderly do not appear to be ideal stool donors. A study by Anand et al., however, did not demonstrate significant difference in alfa diversity between groups above and below age of 60 [180].

**Inflammatory bowel disease (IBD)** is a chronic inflammatory condition of the GI system which encompasses two main types: Crohn's disease (CD) and ulcerative colitis (UC) [181]. Elderly patients with IBD are defined as patients above age of 60 and includes both those who developed disease at a younger age and transitioned to older age and those who developed the disease or are diagnosed for the first time after the age of 60 [182, 183]. For a long time, it was believed that IBD is a disease of young people, yet the prevalence of IBD in patients above 60 is 10-30% [182]. Up to 15% of newly diagnosed patients in the US are elderly, although, the incidence is probably underestimated due to challenges diagnosing this disease in the elderly [184]. Older adult onset UC is more common than CD [185, 186]. The incidence rate of IBD is higher in the US when compared to Asia [185–187], although the incidence is on the rise in Asian countries. It is expected that the prevalence of IBD in elderly will significantly increase due to its relatively low mortality and the fact that majority of those diagnosed at younger age will transition to elderly.

IBD arises in genetically predisposed individuals who develop an abnormal immune response to different gut antigens and their by-products. Genetics seems to be less responsible in the development of IBD in elderly population when compared to those diagnosed at younger age. In fact, 16% of patients younger than 17 who are diagnosed with CD have a family history of IBD, compared to only 7% elderly patients [183]. Apart from a decline in the number and function of T and B lymphocytes, age-related immunosenescense is associated with changes in intestinal microbiota increasing the risk for aberrancy of the immune system and developing IBD [120, 188–190]. As previously mentioned, establishing the diagnosis of IBD in the elderly is difficult. Atypical presentation of IBD, presence of multiple co-morbidities, and polypharmacy affecting bowel function all contribute to challenges in establishing the diagnosis. When compared to their younger counterparts, elderly tend to present with isolated colonic inflammation and perianal fistulas. Less frequently they have small bowel disease or upper gastrointestinal tract involvement [183, 187]. Rectal bleeding is more common than diarrhea, abdominal pain, and weight loss in older onset CD. Older onset disease is more frequently associated with inflammatory phenotype unlike younger patients in whom structural and penetrating disease predominates [182]. On the other hand, older onset UC tends to present with more left colon disease, proctitis, and rectal bleeding with abdominal pain being less pronounced [183, 187]. Older onset IBD may be associated with less inflammation, fewer signs of disease, and less progression of disease. A French cohort study found that 92% of older onset CD did not progress over a period of 2 years and only 3-5% of older onset UC patients progressed to extensive disease [183].

IBD treatment aims to induce and maintain remission, prevent and minimize disease related complication, and improve quality of life. It can be particularly challenging in elderly due to polypharmacy and the presence of multiple medical co-morbidities. Additionally, elderly patients have often been excluded from clinical trials, especially ones including immunosuppressive therapy. Mesalamine is first line therapy for mild to moderate disease and is prescribed in 84% of elderly with UC [183]. Topical mesalamine treats disease involving the last 10 cm of rectum and enemas can potentially address disease up to the splenic flexure [191]. In elderly UC patients with proctitis, a combination of oral and topical mesalamine is more effective than either treatment alone. The rates of nonadherence to mesalamine among elderly is 40-60% [192] and are related to pill size, frequency of dosing, and common side effects such as nausea, vomiting, and abdominal pain. Additionally, use of mesalamine in elderly is difficult due to higher prevalence of CKD among elderly who are more likely to develop nephrotoxicity from mesalamine than younger patients and higher rates of fecal incontinence in this age group, limiting the use of suppository forms. Corticosteroids are very effective in establishing, but not maintaining, the remission in moderate to severe IBD [170]. The use is associated with an increased risk of development and/or worsening of osteoporosis, diabetes mellitus, glaucoma, and hypertension, which are all particularly prevalent in older populations. Risk of hip fracture associated with use of corticosteroids is the highest in those above 60. Despite having the same efficacy in elderly as in younger people, immune-modifying agents are underutilized. A retrospective study of IBD patients above age of 65 found that only 6% of them were on 6-mercaptopurine (6-MP) and just 1% on methotrexate [190]. The potential of immune modifying agents to cause adverse events is not increased in elderly when compared to younger patients [193]. The studies on anti-TNF therapy in elderly are conflicting. A study from Italy compared clinical remission rates among those older than 65 with younger counterparts on anti TNF therapy and found that remission rates were 59% in older UC patients and 65% in older CD patients, which were similar to rates in younger adults [194]. A retrospective study from the US, however, found lower response among elderly who were also more likely to stop therapy [195]. The risk of infection appears to be 12% among elderly on anti-TNF therapy [196]. Live active vaccines are generally contraindicated in immunocompromised patients, including those treated with anti-TNF therapy. Interestingly, recent studies showed that live attenuated herpes zoster vaccine may be safe in some elderly with IBD and should be assessed on a case-by-case basis [196].  Table 5 summarize disease of large intestine in elderly.

## 7. Conclusion

The management of elderly individuals who suffer from GI disease possesses a unique challenge. Clinicians involved in management of this patient population, including internists, family medicine physicians, geriatricians, and gastroenterologist, should be familiar with unique characteristics of this patient population.

It is frequently confusing which changes in GI function represent a part of normal aging processes and which of them are pathological results of a disease process. There is a relative lack of research on this topic and available literature is commonly conflicting. The management of GI diseases in elderly, including diagnostic algorithm as well as therapeutic intervention, is further complicated by frequent presence of comorbidities, polypharmacy, and a limited life expectancy. The elderly commonly have atypical presentation of a disease with more subtle symptoms; hence physicians who are not familiar with these might miss the opportunity to make a diagnosis in timely manner. Polypharmacy and medication side effects further contribute to the complexity of the clinical picture and can derail treating physicians in the wrong direction. Additionally, polypharmacy and comorbidities predispose elderly patients to a more complicated clinical course and increase the probability for development of complications. It is equally important to discuss the goals of care with elderly so our diagnostic and therapeutic interventions align with their expectations.

In summary, our elderly patients represent a specific population with unique needs in regards to diagnostic and therapeutic approaches. Further deepening of our knowledge is necessary in order for us to be able to provide better evidence based and cost effective care in order to maximize the quality of life of this most vulnerable patient population.

## Figures and Tables

**Table 1:** Oral cavity disease characteristics in older adults.

Xerostomia	(i) One of the most common oral sensorial complaints in elderly (ii) Associated with age-related alteration in saliva composition (iii) Most commonly caused by tricyclic antidepressant and anti-Parkinsonian medications

Dysgeusia and ageusia	(i) Commonly due to medication side effects (lithium, metronidazole) (ii) Can result from zinc deficiency or Bell's palsy

Oropharyngeal dysphagia	(i) Common in elderly secondary to stroke, multiple sclerosis, dementia, Parkinson's disease (ii) 50% of nursing home-residents affected (iii) Most common symptoms include cough with swallowing and nasal regurgitation

**Table 2:** Esophageal disease characteristics in older adults.

General remarks	(i) Presbyesophagus in its original meaning doesn't exist (ii) Esophageal problems in elderly more related to comorbidities than to esophagus itself (iii) Studies on esophageal motility yielded conflicting results (iv) Esophageal intrinsic enteric neurons may be more vulnerable to age-related changes than other parts of the enteric system

Esophageal dysphagia	(i) Mechanical causes ( tumor) (ii) Neuromuscular causes ( achalasia, scleroderma) (iii) Inflammatory causes ( eosinophilic esophagitis) (iv) Infectious causes ( candidiasis)

Odynophagia	(i) Most common cause is infectious (ii) Elderly have higher threshold for pain sensation which leads to delayed presentation

GERD	(i) High prevalence among elderly (23%) (ii) Complications more common in elderly (iii) Atypical presentation of odynophagia and dysphagia rather than heartburn (iv) PPI side effects should be kept in mind and lifelong PPI therapy should be avoided (v) Laparoscopic anti-reflux surgery is safe and effective in elderly

Barrett's CLE	(i) More prevalent among elderly (ii) Low grade dysplasia occurs independent of age (iii) Increased age is a risk factor for high grade dysplasia and adenocarcinoma development (iv) Case by case decision about surveillance endoscopy considering life expectancy, co-morbidities and potential harms of surveillance

Pill esophagitis	(i) More prevalent in elderly (ii) Patients with cardiomegaly are particularly high-risk (iii) Female gender and presence of diabetes are independent risk factors (iv) Most commonly presents as chest pain (v) Ulcers discovered on endoscopy in more than 80% (vi) Most common occurs with NSAIDs, aspirin and bisphosphonates

NSAID- non steroidal anti-inflammatory drug.

**Table 3:** Gastric disease characteristics in older adults.

Chronic atrophic gastritis	(i) More prevalent in elderly (ii) Elderly from Japan and China have increased prevalence above 50% (iii) Changes in human gastric microbiota associated with increased risk for gastric cancer (iv) Associated with osteoporosis and autoimmune thyroid disease

H.pylori infection	(i) Incidence in elderly highest in developed countries (ii) Type I cancerogen associated with gastric adenocarcinoma (iii) Extra intestinal manifestation may be more frequent in elderly but association with H.pylori warrants further study

Peptic ulcer disease	(i) Mortality higher than in younger groups (ii) Use of NSAID/aspirin is the main risk (iii) Age-related physiological changes such as reduced gastric blood flow and decreased production of bicarbonates, mucin prostaglandins contribute to higher prevalence (iv) Clinical manifestation is usually atypical (only 30% have typical epigastric pain) (v) Perforation usually lacks typical clinical symptoms of chemical peritonitis (vi) PPI associated with CDC, CAP and OP

PPI- proton pump inhibitor; CDC- Clostridium difficile colitis; CAP- community acquired pneumonia; OP-osteoporosis.

**Table 4:** Small bowel disease characteristics in older adults.

Celiac disease	(i) Typical symptoms frequently absent in elderly, leading to diagnostic delay (ii) Incidence increasing among elderly in North America (iii) After age 65, incidence in women starts decreasing and in men starts increasing (iv) 80% of elderly with CD have IDA at the time of diagnosis (v) Common manifestations include calcium and vitamin D deficiency (vi) CD related neurologic manifestations such as dementia, celiac ataxia and neuropathy are difficult to diagnose and frequently misdiagnosed

Mesenteric ischemia	(i) Atherosclerotic etiology is more prevalent than embolic in elderly (ii) Elderly particularly vulnerable to non occlusive mesenteric ischemia during hypo-perfusion states such as shock and during CPR (iii) CMI presents as post-prandial angina (iv) Elderly with AMI more commonly have confusion, tachypnea, vomiting and diarrhea than classically described abdominal pain out of proportion of physical exam

Small bowel bleeding	(i) Most common etiologies include angiodysplasia and small bowel ulcers (ii) AS and ESRD associated with increase in SB angiodysplasia (iii) SB ulcers associated with NSAID use (iv) Respond favorably to endoscopic treatment

Small intestinal bacterial overgrowth	(i) Very common in elderly (ii) Associated with secondary nutritional deficiency due to malabsorption (iii) Prevalence varies between countries and geographic locations (iv) Higher prevalence in elderly mainly due to achlorhydria and small bowel dysmotility (v) Clinical signs are usually vague and subtle, such as bloating and nonspecific abdominal discomfort

CD- celiac disease; IDA- iron deficiency anemia;ESRD- end stage renal disease;SB-small bowel; CMI- chronic mesenteric ischemia;AMI-acute mesenteric ischemia;CPR-cardiopulmonary resuscitation.

**Table 5:** Large intestine disease characteristics in older adults.

Constipation	(i) Affects 50% of elderly nursing home residents (ii) Caused by decreased mobility, cognitive impairment, co-morbidities and polypharmacy (iii) Not typically caused by decreased colon transition time (if any) (iv) Distinguished from IBS by the lack of abdominal pain and discomfort (v) Stool impaction is a significant complication

Diverticular disease	(i) Most common disease affecting large intestine in elderly (ii) Prevalence increases with age (iii) Prevalence 70% among elderly in the US (iv) Pathophysiology linked to age-related changes of intestinal microbiota and connective tissue of the colonic wall (v) Diverticulitis less likely to become complicated in elderly when compared with younger populations (vi) CCB and statins have protective effect

Irritable bowel syndrome	(i) Prevalence similar across age groups (ii) Elderly more likely to have an organic GI disease, hence, thorough work up recommended (iii) Rectal bleeding is common (iv) Chronic prostatitis can mimic IBS in older men

Clostridium difficile colitis	(i) Elderly are particularly vulnerable (ii) Most severe form of CDC disproportionally affects older nursing home residents (iii) Hospitalization, healthcare acquisition and the recurrence of CDC significantly higher in those above 65 years (iv) FMT failure rate more common in elderly (v) Elderly are not ideal stool donors due to age-related change in intestinal microbiota

Inflammatory bowel disease	(i) 15% of newly diagnosed patients are above 65 years (ii) Age-related immunosenescence and changes in intestinal microbiota predispose to development of IBD (iii) Older CD patients tend to present with isolated colonic inflammation and perianal fistula (iv) Rectal bleeding more common than diarrhea, weight loss and abdominal pain (v) Inflammatory phenotype predominates (vi) Disease less aggressive and progresses more slowly (vii) Mesalamine adherence is poor (40-60% of patients report nonadherence due to pills size, frequency of dosing and GI side effects) (viii) Side effects from immune-modifying agents not increased in elderly when compared to younger counterparts

IBS-irritable bowel syndrome; IBD-inflammatory bowel disease; FMT-fecal microbiota transplantation; CCB-calcium channel blockers.
